# Review of the technology used for structural characterization of the GMO genome using NGS data

**DOI:** 10.1186/s44342-024-00016-1

**Published:** 2024-10-02

**Authors:** Kahee Moon, Prakash Basnet, Taeyoung Um, Ik-Young Choi

**Affiliations:** 1 Department of Agriculture and Life Industry, Kangwon National University, Chuncheon, South Korea; 2 Department of Smart Farm and Agricultural Industry, Kangwon National University, Chuncheon, South Korea

**Keywords:** NGS, Southern blot, GMO, Molecular characterization evaluation

## Abstract

The molecular characterization of genetically modified organisms (GMOs) is essential for ensuring safety and gaining regulatory approval for commercialization. According to CODEX standards, this characterization involves evaluating the presence of introduced genes, insertion sites, copy number, and nucleotide sequence structure. Advances in technology have led to the increased use of next-generation sequencing (NGS) over traditional methods such as Southern blotting. While both methods provide high reproducibility and accuracy, Southern blotting is labor-intensive and time-consuming due to the need for repetitive probe design and analyses for each target, resulting in low throughput. Conversely, NGS facilitates rapid and comprehensive analysis by mapping whole-genome sequencing (WGS) data to plasmid sequences, accurately identifying T-DNA insertion sites and flanking regions. This advantage allows for efficient detection of T-DNA presence, copy number, and unintended gene insertions without additional probe work. This paper reviews the current status of GMO genome characterization using NGS and proposes more efficient strategies for this purpose.

## Introduction

### Structure of the GMO genome

Genetically modified organisms (GMOs) are referred to as living modified organisms (LMOs) and are defined as “newly developed organisms by a new combination of genetic materials or the injection of nucleic acids into cells” by the “Act on the Transboundary Movement of LMO (GMO Act)” from the Domestic Implementation Corporation of The Cartagena Protocol on Biosafety [5]. The Cartagena Protocol is significant because it provides an international framework for biosafety, aiming to protect biological diversity from potential risks posed by GMOs. The genetic material of these organisms has been artificially recombined, through actions including the insertion, deletion, or modification of genes. In transgenic eukaryotes, the process and characteristics of T-DNA insertion involve cloning the target gene (DNA) into a plasmid and inserting it into the genome using *Agrobacterium* during experimentation [2, 15, 22]. Since the first genetically modified (GM) plant was developed using antibiotic-resistant tobacco plants in 1983, many GMO crops, including tomatoes, corn, rice, soybeans, cotton, and rapeseed, have been developed via transgenic biotechnology [7, 29, 32]. During this process, one or more T-DNAs can be inserted into multiple sites. Similarly, in transgenic prokaryotes, the target gene (DNA) is cloned and inserted into a plasmid and then inserted into the desired prokaryote for development during experimentation. Depending on the research purpose, the plasmid can be inserted into the recombinant plasmid or the prokaryotic genome. To use them for commercial purposes such as food and feed, GMOs developed through biotechnology must undergo safety testing for human and environmental risks. The safety test standards for GMOs in each country are separately established and performed in accordance with the international GMO safety standards CODEX and EFSA’s biosafety assessment standards.

### Assessment criteria and technologies for determining the LMO genome structure for commercialization vary by country

The Codex Alimentarius Commission, an international organization established to develop and promote food safety standards and guidelines, formulated the “Guideline for the Conduct of Food Safety Assessment of Foods Derived from Recombinant-DNA Plants” (CAC/GL 45–2003). Molecular characterization of a genotype contributes to a thorough evaluation of the potential impacts of recombinant DNA plants on food, feed, and environmental safety [34]. According to CODEX guidelines, the following information must be provided to characterize genetic modifications in plant genomes: (a) characterization and description of the introduced genetic material (inserted DNA), (b) the number of insertion sites; (c) the organization of the inserted genetic material at each insertion site, including copy number and sequence data of the insertion and surrounding regions, sufficient to identify any substances expressed as a result of the inserted material as well as other information such as analysis of transcripts or expression products to identify any novel substances that may be present in the food; and (d) identification of any open reading frames (ORFs) within the inserted DNA or contiguous plant genomic DNA that could result in fusion proteins [6]. Each country develops and implements assessment standards in accordance with these guidelines, ensuring that they align with national regulations.

The European Food Safety Authority (EFSA) requires molecular characterization of the DNA sequences inserted into the genome of genetically modified plants as part of the risk assessment process. This requirement is detailed in Regulation (EU) No. 503/2013 and the EFSA guidance on the risk assessment of food and feed from genetically modified plants [9]. EFSA specifies the use of next-generation sequencing (NGS) or Sanger sequencing for sequencing the introduced DNA and its flanking regions. These methods require the identification of insertion sites, copy numbers, and genetic stability across generations [10, 11].

The United States Department of Agriculture’s (USDA) Animal and Plant Health Inspection Service (APHIS) regulates most plants developed using recombinant DNA technology for commercial purposes under 7 CFR part 340. These plants are considered regulated articles. If genetic material is inserted, the nucleotide sequence of the inserted genetic material must be reported. Applicants must provide the nucleotide sequence in FASTA format or other acceptable formats (e.g., GFF, MS Word). The annotation of the inserted genetic material must include the nucleotide position and the name of the inserted component [45].

The Canadian Food Inspection Agency (CFIA) has issued industry guidance on the use of whole-genome sequencing (WGS) for premarket submissions of LMO plants based on discussions within the Canada-US-Mexico Trilateral Technical Working Group (TTWG). Although traditional molecular biology methods such as Southern blotting, Sanger sequencing, and polymerase chain reaction (PCR) remain acceptable, CFIA also permits the generation of data via advanced technologies such as high-throughput sequencing, next-generation sequencing (NGS), or whole-genome sequencing (WGS). According to CFIA [4], the criteria for molecular characterization assessment include “A) The DNA that was inserted, deleted, or modified; B) The number of complete or partial copies of the inserted DNA; C) The organization of any inserted or altered genetic elements, including coding, regulatory, and other non-coding regions; this may include sequence data of the inserted DNA and surrounding regions where appropriate (e.g., to characterize a partial insertion or rearrangement); D) The mode of inheritance and stability of the genetic changes.”

Food Standards Australia New Zealand (FSANZ), the agency responsible for developing food standards in Australia, issued the Application Handbook in 2019, which outlines the approval criteria for the molecular characterization of new LMOs produced using genetic technology. According to FSANZ [12], the following information must be included: “A) Identification of all transferred genetic material and whether it has undergone any rearrangements,B) Determination of the number of insertion sites and the number of copies at each insertion site; C) Full DNA sequence of each insertion site, including junction regions with the host DNA; D) A map depicting the organization of the inserted genetic material at each insertion site; E) Details of an analysis of the insert and junction regions for the occurrence of any open reading frames (ORFs).”

The Ministry of Agriculture, Forestry and Fisheries (MAFF) of Japan regulates the assessment of human health risks associated with genetically modified organisms (GMOs) by specifying the following requirements for the presence and stability of introduced DNA within cells: (A) the location of the introduced nucleic acid replication product (differentiating between chromosomes, intracellular organelles, and extrachromosomal elements); B) the number of copies of the introduced nucleic acid replication product and its stability across multiple generations; and C) when multiple copies are present on chromosomes, whether they are adjacent or separate [28].

The Taiwan Food and Drug Administration (Taiwan FDA) requires molecular characterization data to determine gene insertion sites and copy numbers. Experimental methods such as Southern blotting or sequencing analysis can be used. If DNA sequences are provided, they must include the flanking regions of the gene. The insertion site must be clearly indicated, and the copy number of the flanking sequences in the original organism must be provided [43].

The Ministry of Food and Drug Safety (MFDS) in Korea has established five criteria for evaluating information regarding inserted genes in genetically modified agricultural, livestock, and fishery products. These criteria are as follows: (A) the characteristics and functions of the genes inserted into the genome of genetically modified agricultural, livestock, and fisheries products, (B) the number of insertion sites, (C) the composition of the inserted genes at each insertion site, (D) the presence of open reading frames (ORFs) within the inserted genes and adjacent host genome genes and their transcriptional and expression potential, and (E) information related to genetic stability. To fulfill these requirements, next-generation sequencing (NGS) data may be submitted as an alternative to Southern blot analysis data [31]. Currently, the MFDS is the only regulatory body in Korea that approves NGS data, whereas other organizations, such as the Rural Development Administration and the Korea Disease Control and Prevention Agency, have not yet approved their use [31].

A total of 42 countries, including 16 individual nations and 26 EU member states, import LM crops for food, feed, and processing purposes. Additionally, 72 countries worldwide have adopted LM crops [16]. The evaluation criteria for the genomic structure of LMOs for commercialization in each country are based on the standard requirements outlined by organizations such as CODEX. Applicants are required to provide experimental data to demonstrate compliance with these standards. The commonly requested molecular characterization information includes the presence of introduced genes, their locations, their copy numbers, and their nucleotide sequence structure.

There is an increasing trend among countries to accept or recognize whole-genome sequencing (WGS) or next-generation sequencing (NGS) data either as replacements for or in addition to traditional molecular characterization methods such as Southern blotting and PCR. The United States was the first to accept NGS data in 2012, followed by Canada and Japan in 2014. Most recently, China recognized NGS data in 2023. Currently, NGS data are accepted by a total of 18 countries (Table 1).

**Table 1:** List of countries and GMOs with approved WGS data by year

Year	Country	Approved GMOs	Cumulative total GMOs
2012	USA	1	1
2014	Canada, Japan	2	3
2015	Australia, Mexico	2	5
2016	Argentina, Brazil, Colombia	3	8
2018	Indonesia, Philippines, Singapore, South Africa	4	12
2019	European Unit, Paraguay	2	14
2020	Vietnam	1	15
2021	Thailand, Uruguay	2	17
2023	China	1	18

### Comparative analysis of LMO genomic structures using different methods

#### Traditional methods

**Fig. 1:**
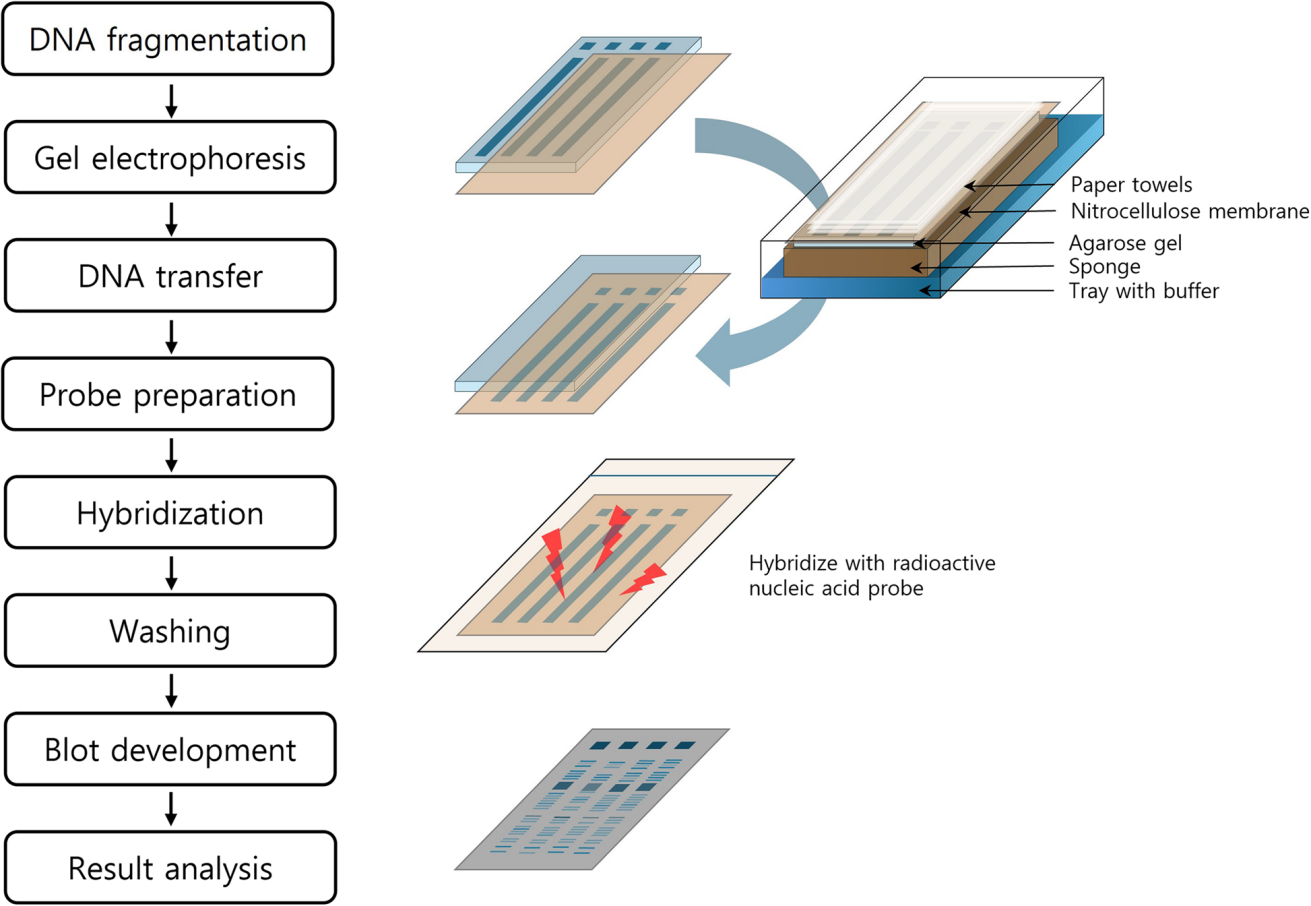
Flow chart of Southern blot hybridization

Southern blotting is a technique developed by Southern in 1975 to analyze the presence of specific genes in genomic DNA [42]. The basic principle involves using the complementary binding characteristics of DNA (hybridization), in which a single-stranded nucleic acid forms a double helix with another complementary strand under specific conditions to determine the presence of specific nucleotide sequences in the DNA (Fig. 1). The experimental procedure is as follows:

A) DNA fragmentation: Genomic DNA is extracted from the sample and fragmented using restriction enzymes.
B) Gel electrophoresis: The fragmented DNA is separated by size through gel electrophoresis.
C) DNA transfer: Using capillary action, the DNA in the electrophoresis gel is transferred to a positively charged nitrocellulose membrane.
D) Probe preparation: A specific nucleotide sequence is amplified using dNTPs (A, T, G, and C) labeled with a radioactive isotope to create a probe.
E) Hybridization: The membrane with the adsorbed DNA is incubated with the radioactive probe at a specific temperature (Tm) to induce complementary binding.
F) Washing: Nonspecifically bound proteins are removed by washing to maintain only specific binding between the DNA and the probe.
G) Expose and develop: The membrane with the specific DNA-probe binding is exposed to X-ray film for a specific time period, followed by the development of the X-ray film.
H) Results analysis: The developed X-ray film was analyzed to determine the presence of specific nucleotide sequences.

#### Next-generation sequencing (NGS)

Next-generation sequencing (NGS) enables the rapid analysis of hundreds to thousands of genes or entire genomes within a short time frame [40]. It is used for DNA and RNA sequencing as well as for detecting variations and mutations [36]. The genomic DNA of LMOs can be fragmented using a shotgun approach and then sequenced using NGS equipment. This approach allows for the analysis of LMO characteristics, such as the presence, copy number, and nucleotide sequence of the inserted DNA at each insertion site [23, 49, 50]. The verification process using NGS proceeded as follows: Illumina sequencing reads were generated, followed by mapping to the plasmid backbone, isolation of the flanking region, surveys of the junction region reads, and, lastly, confirmation through PCR validation [8].

**Fig. 2:**
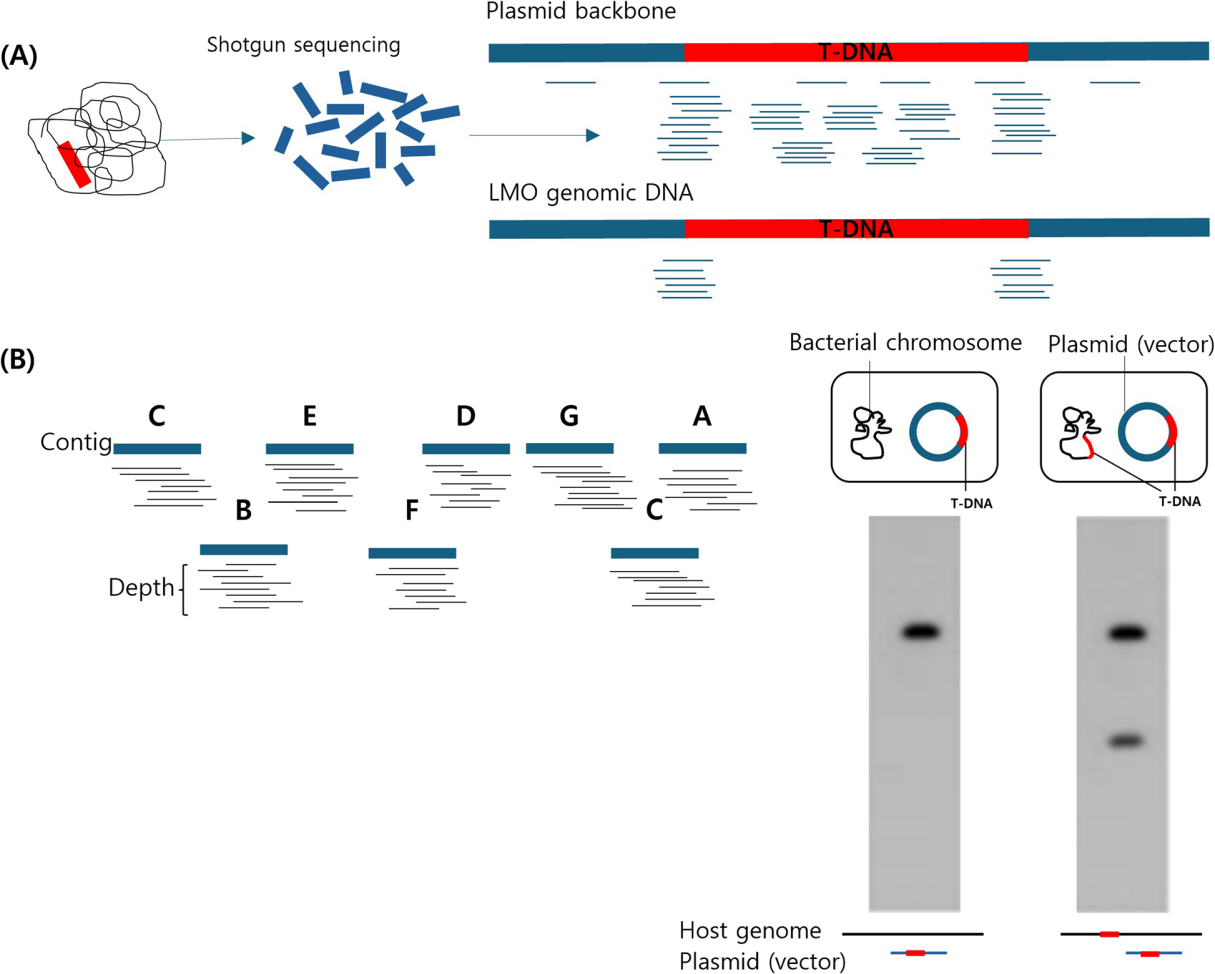
Comparison of NGS techniques for evaluating the genome structure of Eukaryotes and Prokaryotes. **A** NGS analysis techniques for evaluating the genome characteristics of eukaryotic LMOs: to assess the genome characteristics of eukaryotic LMOs, a shotgun library was created from the whole genome of the event and sequenced. Mapping these sequences to the plasmid backbone revealed integration sites where T-DNA was exclusively attached. **B** NGS analysis techniques for evaluating the genome characteristics of prokaryotic LMOs: • De novo assembly (left): During de novo assembly, contigs are formed, allowing for the confirmation of sequence depth at each locus. • Genome structure evaluation (right): This method distinguishes between scenarios in which T-DNA is integrated solely into the plasmid and cases in which it integrates into both the plasmid and the bacterial chromosome. Confirmation of the genome structure involves analyzing the PCR results and the composition of the genome

For the identification of introduced and unintended DNA in eukaryotes, the whole genome is fragmented to create a shotgun library and then sequenced. The NGS data are then mapped to the recombinant plasmid backbone to identify the regions where the backbone is mapped. The copy number and sequence information at each insertion site can be determined by investigating fragments mapped to the T-DNA flanking region of the plasmid backbone and aligning them with non-GMO genome references used in LMO development. Verification of the inserted DNA sequence structure is possible through independent verification of the sequence in that region. The adjacent nucleotide sequences of the inserted gene can be confirmed by PCR amplification of the T-DNA insertion site in the LMO genome, followed by sequence analysis (Fig. 2A) [35].

For prokaryotes, the identification of unintended introduced DNA can be performed using contigs generated from de novo assembly of NGS data. The copy number and sequence information at each insertion site can be determined by examining the nucleotide sequences of de novo assembled contigs. Verification of the inserted DNA sequence structure can be achieved through independent sequence analysis of the region. The adjacent nucleotide sequences of the inserted genes were confirmed by PCR amplification of the T-DNA insertion site in the LMO genome, followed by sequence analysis (Fig. 2B) [47].

### Challenges in evaluating the GMO genome structure

The molecular characterization of genetically modified (GM) plants before commercial release is a crucial step for assessing their safety and obtaining regulatory approval [6]. Molecular characterization is necessary for the commercialization of LMOs. Southern blot analysis, which uses sequence-specific probes homologous to the introduced genes, has been widely used for the molecular characterization of transformation events to determine the presence and copy number of introduced genes [42, 50]. Additionally, methods such as PCR and Sanger sequencing [33, 48] or microarray analysis [24] can be used to detect transgenes.

However, these methods, especially Southern blotting, require skilled techniques, are time-consuming, involve safety issues related to the use of isotopes, and necessitate the establishment of conditions for each experiment due to variations in the nucleotide sequence of the target sequence. Moreover, there are limitations in detecting single-nucleotide polymorphisms (SNPs) or small insertions and deletions within the T-DNA and its insertion site [19].

### The purpose of the study

This study was to analyze and review the current status of NGS data use for the characterization of genetically modified organism (GMO) genomes and to propose more efficient strategies for evaluating GMO genome characteristics using NGS.

## Materials and methods

### Material

Using NGS for molecular characterization involves analyzing the whole-genome sequence (WGS) of GMO samples. The setup of a library is the initial step in this procedure. Genomic DNA is extracted from the sample seeds and then fragmented into approximately 500-bp fragments. Each fragment is tagged to create a unique library.

Initially, the sample must undergo cleanup to prevent contamination from external sequences. Tissue and seed samples should remain uncontaminated. The experiments should be conducted in a molecular biology laboratory to prevent contamination from environmental or bacterial backbones. Even when grinding dried samples, caution is necessary. The next-generation sequencing (NGS) data are digital and not analog and are represented by bands on a gel, and contamination from bacteria or product crossover can occur, resulting in identity issues. The sample is ground into a fine powder, and DNA is extracted for quantification.

### High-throughput DNA sequencing

Genomic DNA is fragmented, and 3′-5′ exonuclease is used to remove 3′ overhangs, while polymerase fills in 5′ overhangs and repairs DNA ends. After AMP cleanup, a single “A” is added to the 3′ end for DNA 3′ end adenylation, preventing blunt-end ligation and preparing for adapter ligation in the next step. Multiple indexed adapters with sequencing flow cell DNA hybridization are attached to the ends of the fragments. DNA fragments with attached adapters are amplified by PCR or similar techniques to create additional libraries. The sample is concentrated to remove excess adapters, erroneous DNA, or other impurities from the PCR, and the library concentration is adjusted. The fragments are primarily extracted at a size of 500 bp during this process.

The major sequencing platforms used for generating reads at both ends of each fragment in library sequencing are Illumina, Thermo Fisher Ion Torrent, Pacific Biosciences (PacBio), and Oxford Nanopore. Currently, NGS technology cannot produce complete genome sequences; instead, the raw data generated by NGS devices represent relatively short reads that are fragments of the organism’s genome. For long-read sequencing, useful platforms include PacBio’s Sequel and Oxford Nanopore’s MinION devices. Short-read sequencing technologies include Illumina’s iSeq 100 and MiSeq as well as Thermo Fisher’s Ion Torrent.

These sequencing devices detect signals representing the nucleotide sequence and undergo a conversion into readable nucleotide sequences by a computer. The sequencing analysis process includes quality control measures, and raw data are generated along with quality scores indicating the reliability of sequence analysis for each base pair of reads. This protocol is explained in greater depth in the “Comparative assessment of detailed techniques using NGS technology for evaluating the genome characteristics of GMOs.”

### Sequence data analysis

**Fig. 3:**
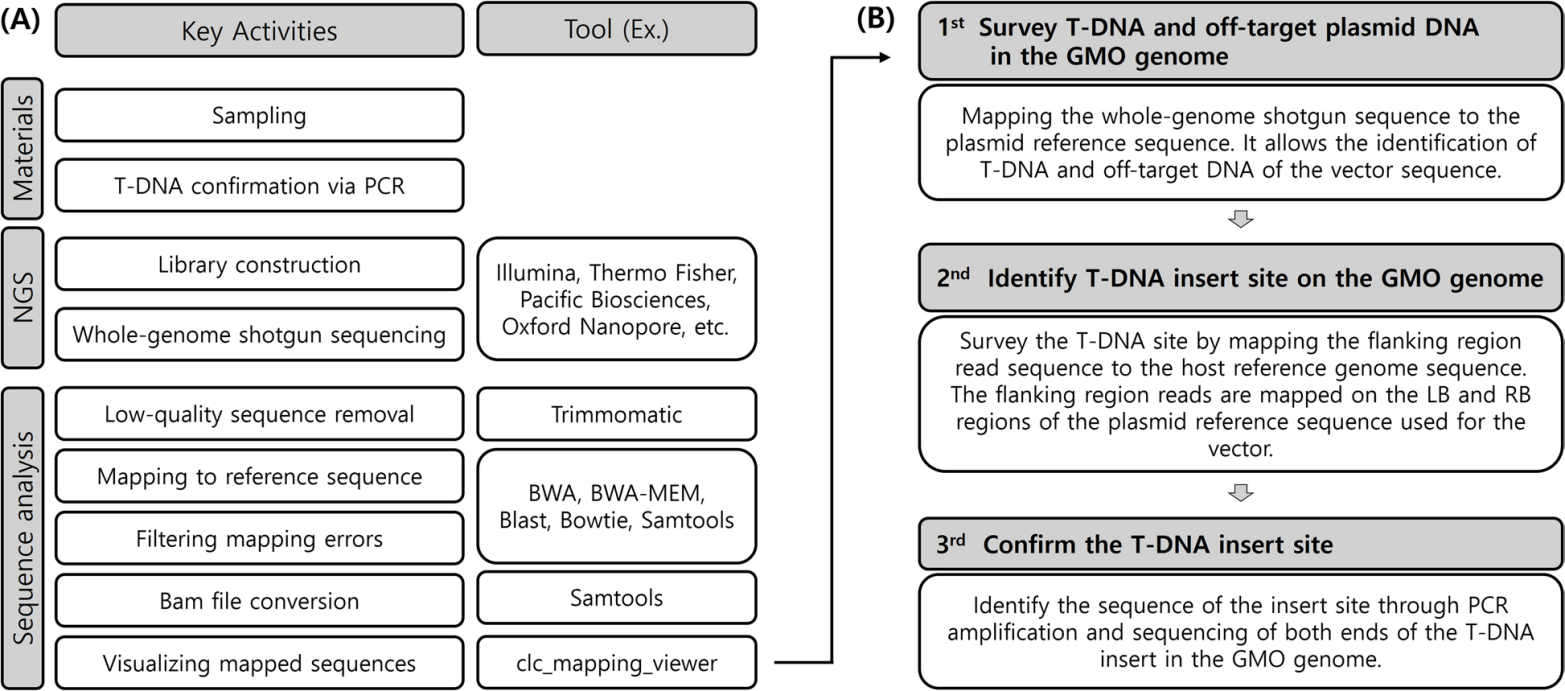
The workflow of the NGS strategy to characterize GMO genome structure. Key activities and tools for NGS data analysis at each stage (**A**). The strategy to characterize T-DNA identification using NGS data (**B**)

Biological informatics analysis is performed using the latest databases for conducting both intra- and interspecies similarity searches to represent standardized electronic format sequence information for both 5′ and 3′ adjacent regions at each insertion site (EU No. 503/2013). Alignment software such as BLAST, Bowtie, BWA, and BWA-MEM is used with plasmid backbone sequences as references to map high-quality flanking reads of the right border (RB) and left border (LB) of the inserted recombinant gene. Following this bioinformatics analysis, visualization software is used to render the mapped reads. Through this visualization process, conclusions about molecular characteristics, such as T-DNA identification and detection of unintended insertion genes, can be rapidly interpreted. For evaluation purposes, accurate versions must be provided if general biological informatics software such as BLAST and read filtering or trimming tools are used. Each tool contains multiple parameters and options, so potential issues should be flagged, and parameters and options should be accurately represented with their justification, for transparency assurance (Fig. 3).

After testing various combinations of alignment software, BWA-MEM, which provides both end-to-end and local or chimeric alignment, demonstrated the best performance with the highest accuracy and a relatively short computation time. Although BWA-MEM may have a longer runtime than the Bowtie or BWA-ALN algorithms, it has been shown to generate more accurate and reliable results [35].

## Results

### Advantages and disadvantages of traditional methods and NGS technology for evaluating the structural characteristics of GMO genomes

The evaluation criteria for the structural characteristics of GMO genomes, such as the presence of T-DNA, its insertion site, its nucleotide sequence structure, its copy number, and the presence of unintended inserted DNA, can yield similar conclusions using both Southern blot and NGS technologies [13, 14, 19, 23, 49]. Both methods offer high reproducibility and provide accurate results. However, Southern blot experiments require different designs depending on the event, and the procedure is labor intensive, with manual analysis tools, resulting in lower throughput and longer processing times. These traits contrast with NGS methods, which benefit from automated software tools and standardized designs, resulting in significantly shorter analysis times and higher throughput (Table 2).

**Table 2:** Comparison of the key features of Southern blots and NGS

Columns		Southern blot	NGS
Key characteristics	Experimental design	• Event specific	• Standard
	Procedure	• Labor-intensive	• High throughput
	Reproducibility	• High but multiple manual procedures may cause variation in the results	• High
	Data analysis tools	• Manual	• Automated software tools for data analysis
	Data output	• Blot images • DNA fragment level of details	• Mapping/visualization of sequence reads • Nucleotide level of details
	Accuracy	• High in combination with Sanger sequence	• High
	Throughput	• Low	• High
Molecular characterization	Presence/absence of T-DNA	• Confirmation through genome digestion with restriction enzymes and analysis with T-DNA probe, validated by band presence	• Confirming mapping regions by aligning the entire nucleotide sequence reads to the plasmid backbone
	Insertion site	• Determination through PCR primer walking using T-DNA primers	• Confirmation by mapping plasmid flanking region reads to the genome reference
	Sequence structure	• Separate analysis of nucleotide sequences within the T-DNA region	• Confirmation of sequence structure by mapping genomic position and T-DNA reads to the plasmid
	Replication number	• Determination via count of Southern bands	• Verification through reference mapping using reads from plasmid backbone flanking regions
	Unintended inserted DNA	• Inability to confirm using only T-DNA as a probe • Probing of the entire plasmid for confirmation	• Detection possible during mapping to the plasmid backbone
Endpoint verification	Copy number and integrity	• Confirming copy number based on the pattern of bands detected using specific probes and the size of fragments	• Verifying copy number and integrity of insertions by examining the number of reads and junctions matching the T-DNA region indicated on the map
	Absence of backbone	• Confirming the absence of backbone insertion by detecting bands using specific probes at the respective positions	• Verifying the absence of backbone insertion by observing the absence of read sequences in the backbone region of the same plasmid map where T-DNA has been confirmed
	Stability across generations	• Determining stability by confirming if bands observed in characterized generations are consistently observed at the same positions across multiple generations using specific probes	• Confirming stability by examining consistency in T-DNA read mapping, number of junctions, and the absence of backbone across characterized and multiple generations

**Fig. 4:**
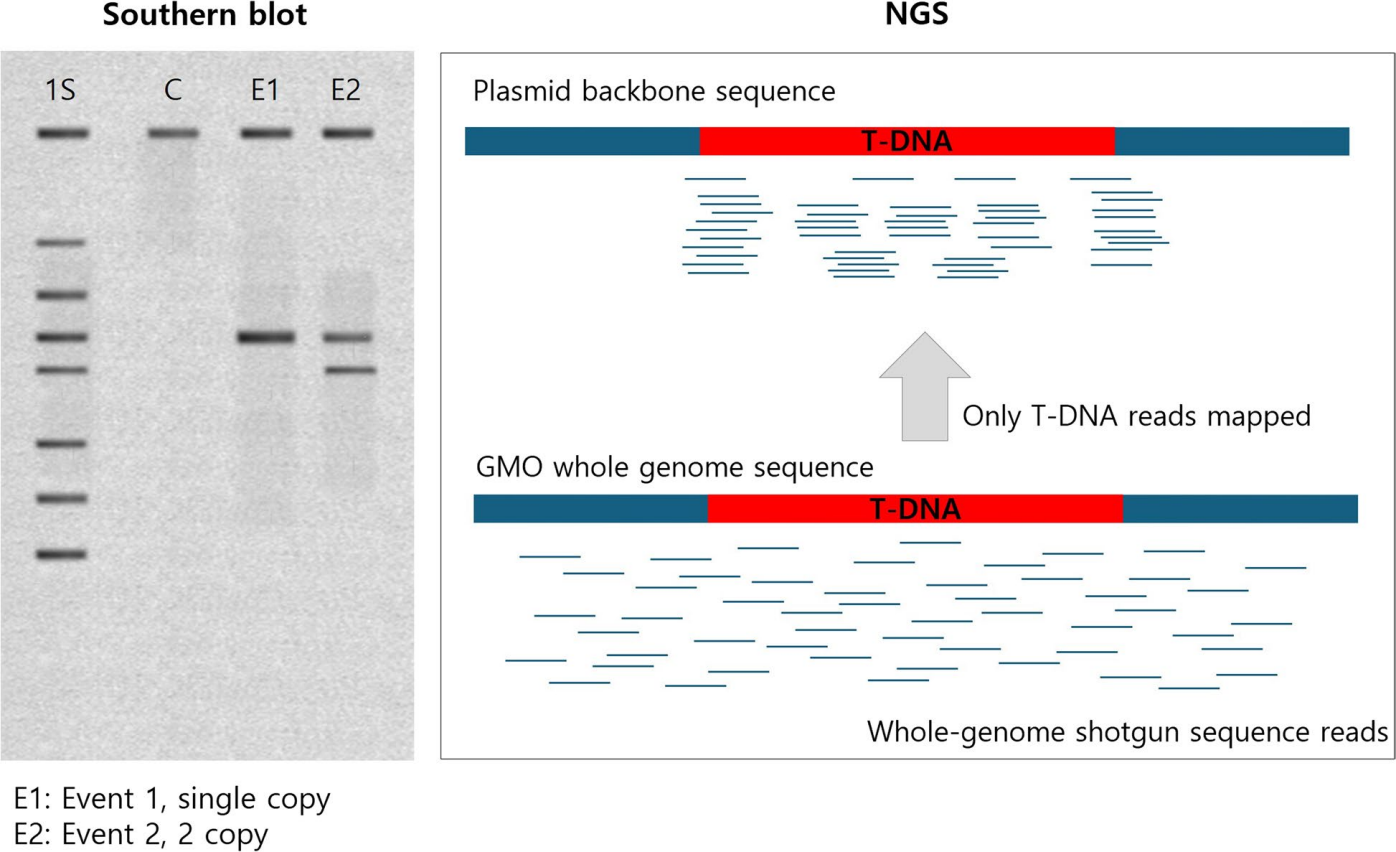
Comparison of evidence for copy number, plasmid insertion-unintended insertion using Southern blotting and NGS

For Southern blot analysis, confirming the presence of T-DNA requires genome digestion with restriction enzymes and T-DNA probe analysis to detect bands. To determine the copy number of the inserted gene, the presence of unintended sequences, and the stability of insertions, using only the T-DNA as a probe is insufficient. Instead, probes spanning the entire transformation plasmid must be designed, hybridized with fragmented DNA, and analyzed for banding patterns via Southern blotting (Fig. 4 left). Additionally, specific backbone probes must be designed and hybridized to confirm the absence of a backbone, which can be inferred from the absence of hybridization. To assess the stability of T-DNA across generations, additional blots with T-DNA-specific probe sets must be generated. Stability can be confirmed by observing consistent band patterns at the same positions across multiple generations using specific probes. Southern blotting involves repeated design and production of probes for each item, followed by manual analysis, making it labor intensive, time-consuming, and less expensive than NGS methods.

NGS does not require the production of probes. Instead, it involves mapping the whole sequence of the plasmid against the WGS dataset of the target analysis. The mapped reads are then rendered and analyzed to obtain visualization data. Without the need for additional data generation for each item, NGS allows of the user to identify the entire T-DNA, copy number, presence of unintended introduced genes, and absence of a backbone using the same mapping and diagram. It enables easy and rapid confirmation of junction sites, replication numbers, and the depth of coverage. With standardized designs, NGS offers high reproducibility and accuracy, as well as high throughput, through automated software tools for data analysis (Fig. 4 right).

### Comparative assessment of detailed techniques using NGS technology for evaluating the genome characteristics of GMOs

To evaluate the genomic characteristics of GMOs (genetically modified organisms) using next-generation sequencing (NGS) technology, sequencing libraries were created, and reads were generated at both ends of each fragment by sequencing the DNA fragments. The major sequencing platforms used here were Illumina, Thermo Fisher Ion Torrent, Pacific Biosciences (PacBio), and Oxford Nanopore. Since NGS devices only generate relatively short reads or portions of an organism’s genome, they are currently unable to provide full genome sequences [17].

Long-read sequencing technology, exemplified by PacBio’s Sequel and Oxford NanoPore’s MinION, is useful for decoding DNA sequences over longer stretches. This technology can generate longer reads, enabling more accurate sequencing and the assembly of long DNA sequences, which significantly reduces the number of contigs formed by repetitive sequences within the genome. Long-read sequencing is advantageous for assembling large genomes or interpreting complex gene structures [21]. For instance, it allows for more precise detection and analysis of large structural variations, insertions/deletions of nucleotides, gene duplication events, etc. [18]. Reading long genes with a single read enables the interpretation of sequence continuity. One of the key advantages of long-read sequencing is its ability to assemble genomes of new species without relying on existing reference sequences. This is particularly useful for studying species genome structure or variants that are not yet well characterized [17, 38]. However, longer reads tend to have higher error rates, impacting sequence quality [21]. Additionally, long-read sequencing is relatively expensive and time-consuming.

Short-read sequencing technology, represented by Illumina’s iSeq 100, MiSeq, and Thermo Fisher’s Ion Torrent, is advantageous for conducting large-scale sequencing projects because it can generate a substantial amount of sequencing data within a single experiment. It has demonstrated high performance in detecting various genetic variations, such as small-sized gene mutations and single-nucleotide polymorphisms (SNPs). Moreover, it offers high accuracy and relatively low-cost analysis. However, it has limitations, such as restricted read lengths, which makes it challenging to assemble long DNA sequences and may limit the detection and analysis of complex variations such as large structural variations or significant insertions/deletions. Assembling long sequences from sequenced reads requires overlapping between reads, and for accurate sequence analysis, a known reference sequence is often necessary, which can make analysis challenging for uncharacterized species. The pros and cons of short-read sequencing technology vary depending on its characteristics, so it should be chosen carefully according to research goals and needs.

The principle of Illumina/Solexa platforms involves cutting DNA fragments and using libraries created by attaching different types of adaptors to both ends. The prepared library is passed through a slide called a flow cell, to which adaptors and complementary oligos are attached, and then amplified. Subsequently, the DNA synthesis process involves measuring the fluorescence emitted as bases are incorporated, using the sequencing by synthesis technique to analyze the nucleotide sequence. The advantage is that the cost per base pair analyzed is very low, while the disadvantage is that the length of the DNA sequence that can be read at once is relatively short (Table 3).

**Table 3:** Comparison of major NGS sequencing platforms for evaluating the genome characteristics of LMOs

Sequencing platform	Illumina		Ion Torrent	PacBio Sequel	Oxford nanopore
	iSeq 100	MiSeq			
Mean read length (bp)	2 × 150	2 × 300	400	10–20 kb	13–20 kb
Data output (Gb)	0.3–1.2	0.3–15	0.6–15	5–10	15–30
Sequencing run time (h)	9–17.5	4–55	~ 19	~ 48	~ 48
Error rate	Low		Low	High	High
Read length	Short		Short	Long	Long
Cost	Low		Low	High	High
Advantage	Low cost, high accuracy		Straightforward preparation, rapid sequencing, low cost	Real-time data generation, rapid result verification	Large genome assembly, complex gene interpretation
Disadvantage	Limited read length, challenging long DNA assembly, complex variation detection limits			High error rates, requires error correction, high costs	High error rates, high costs

^*^Prepared by the authors with reference to Byung-Yong Kim’s “Recent Research Technologies for Quality Control of Commercial Probiotics” (Curr. Top. Lact. Acid Bact. Probiotics. 2019; 5:39–46) and Jackson SA’s “Improving end-user trust in the quality of commercial probiotic products” (Front. Microbiol. 2019; 10:739)

^**^Long-read lengths are typically 10’s of kilobases. Short read lengths are typically 100’s of bases. High error rates are estimated to be greater than 1%, whereas low error rates are less than 0.1%

The Thermo Fisher Ion Torrent NGS instrument employs emulsion PCR for amplification followed by sequencing by synthesis. Instead of using enzymes for signal generation, H + ions generated when each dNTP is incorporated are detected. These H + ions, produced during polymerization, influence a semiconductor chip known as the ion chip, allowing the determination of the sequence of each base by analyzing the pattern of H + ion release. Ultimately, pH changes occur and are detected by sensors [30, 39]. However, this pH variation is not directly proportional to the number of bases being bound, leading to inaccuracies in measuring homopolymer lengths [37]. Nonetheless, the preparation process is relatively straightforward, allowing rapid sequencing at a relatively low cost [44].

The PacBio SMRT (single-molecule real-time) sequencing technology by Pacific Biosciences employs circular DNA templates called SMRTbells. These templates consist of single-stranded hairpin adapters connected to both ends of the double-stranded DNA insert. DNA polymerase is coupled with the SMRTbell template, and the template is loaded into a SMRT cell containing up to eight million zero-mode waveguides (ZMWs) for sequencing. During sequencing, the polymerase incorporates fluorescently labeled dNTPs onto the strand as it passes through the SMRTbell template. When a dNTP is added, a laser excites the fluorophore, which is then recorded by a camera. Subsequently, the fluorophore is cleaved from the nucleotide, and the process repeats thousands of times, revealing the identity and order of the bases [27]. PacBio technology typically generates reads 10’s of kilobases in length, which is significantly longer than those obtained from Illumina sequencing. Its ability to generate sequencing data in real time allows for the rapid verification of the results [38]. However, its disadvantage lies in its relatively higher error rates within reads, necessitating error correction and analysis. Additionally, the complexity of the technology results in relatively higher costs.

ONT (Oxford Nanopore Technologies) long-read sequencing technology employs linear DNA molecules. The sequencing process begins by attaching double-stranded DNA to sequencing adapters with motor proteins. A DNA mixture is loaded into a flow cell containing hundreds to thousands of nanopores. Motor proteins unwind the double-stranded DNA and thread it through the nanopore at a constant speed along with the flow of current. As DNA passes through the nanopore, specific disruptions in the current occur, and they are analyzed in real time to reveal the order of bases on the DNA strand. Furthermore, there have been cases in which reads of over 1 Mb have been generated through ONT sequencing, marking the entry of the genomics community into the realm of megabase-length sequence reads. However, its disadvantages include high error rates and relatively high costs.

To achieve high-quality whole-genome sequencing (WGS), long-read lengths with low error rates should be decoded. However, due to inherent technical differences, it is challenging for a single sequencing platform to achieve complete decoding of the genome. Therefore, it is generally more efficient to mix and analyze data produced by two or more platforms that generate different types of data [21].

### Identification of effective NGS strategies for evaluating the genome characteristics of GMOs

The evaluation criteria for GMO genome characteristics vary by country and department, but common key assessment items include sample information, NGS data information, identification of inserted genes (nucleotide sequences), insertion site, copy number, copy number and nucleotide sequences by location, adjacent nucleotide sequences, and stability across generations. Efficient methods for evaluating GMO genome characteristics using NGS technology are as follows:

Sample information should include details on the GMOs, control groups, and plasmids, with additional information on *Agrobacterium* for plants. Information on the lineage and generation of samples, as well as sampling (selection) methods and timing, including breeding pedigree, should be described. Details on DNA extraction methods, transformation techniques, and other processing technologies should be provided. The samples and results used for DNA extraction, sequencing, and WGS-based data analysis should be identical to the GMO samples under evaluation, and a minimum of three times the amount of sample should be preserved to allow for additional sequencing experiments.

To evaluate the quality of NGS data, various information about NGS data is needed, including the NGS library, the NGS platform, sequence quality, and filtering options for the raw data. The construction of the library involves explanations of the production steps, such as DNA fragmentation methods and fragment selection, with references to relevant papers or websites. For targeted NGS (such as SbS using sequence capture methods), data on the capture efficiency must be provided, demonstrating hybridization of the target sequence with DNA fragments similar to those in Southern blot analysis before NGS analysis. In the case of targeted NGS (SbS), the surrounding genome sequences of the inserted DNA can be determined by Sanger sequencing for comparison with reference sequences. The sequencing platform used for data generation should be specified, including the manufacturer, model, and software version. To ensure sequencing strategy and quality control, reliable data criteria and strategies for evaluating the genomic characteristics of LMOs using NGS should be described. This description includes explanations on the minimum expectations for reliable NGS data and methods for calculating the read depth and average read depth of NGS fragments. For the preparation of a comparison table between raw data and filtered data, information on the quality and characteristics of the raw data produced by the NGS platform must be provided, along with the details of the software, filtering criteria, and results used for filtering the NGS data used to evaluate the LMO genome characteristics.

To identify the inserted genes (T-DNA) and demonstrate coverage across the entire T-DNA region, technical methods and visualization data must be submitted. A coverage graph demonstrating the coverage across the entire T-DNA region should be provided. This graph should indicate the presence of junctions and map sequence reads to compare with the transformation plasmid for visualization. The results should include information such as average depth for the mapping region of the inserted DNA and should be presented in a visual format.

**Fig. 5:**
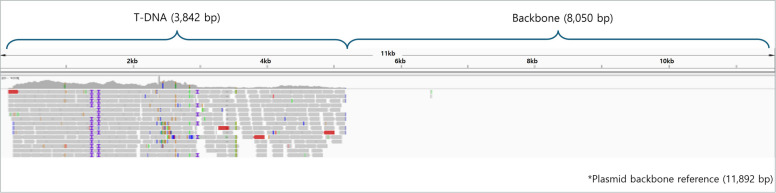
Example image of unintended T-DNA identification using NGS data: mapping results of LMO genome NGS data to the plasmid backbone reference used for transformation. In the nontargeted DNA region, 38 fragments (coverage depth = 0.00x) were mapped but confirmed as missing reads

To confirm the presence of unintended insertions, it is necessary to examine the impact of the insertion. Evidence and explanations regarding the presence or absence of deletions in the host genome gene sequences must be provided as well as the presence of unintended additional sequences and the creation of unintended novel reading frames due to the insertion. When a novel reading frame not present in the host is created, information about proteins with similar amino acid sequences should be provided. Additionally, the presence of vector backbone sequences must be verified. For this purpose, sequence reads should be mapped and compared to the transformation plasmid to visualize the absence of reads in the region corresponding to the transformation plasmid, including the backbone. Evidence demonstrating the absence of reads in the region of the transformation plasmid, including the backbone, should be submitted. If reads are occasionally mapped, the reasons for this occurrence must be investigated (Fig. 5).

**Fig. 6:**
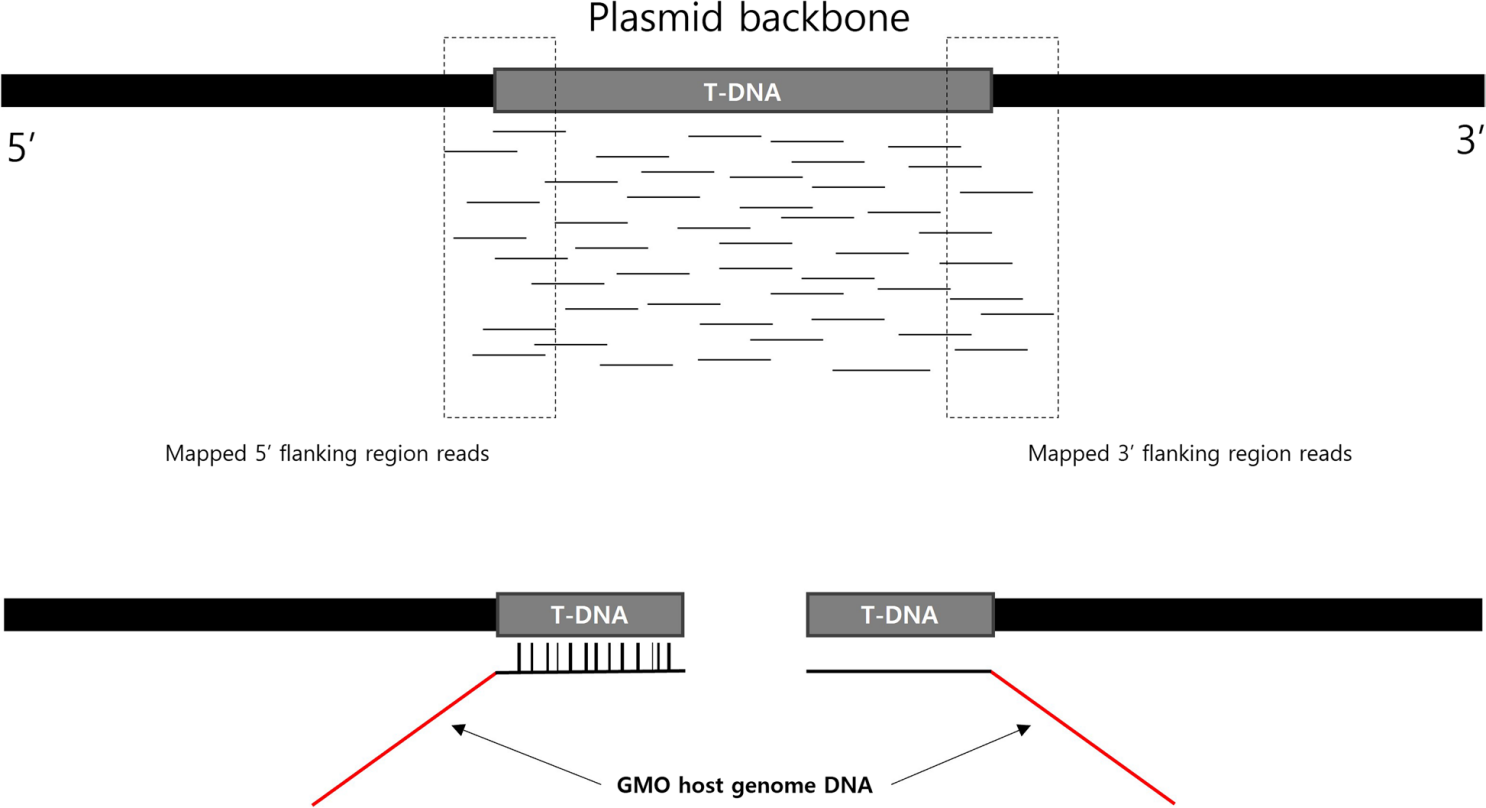
Example of T-DNA localization on the LMO genome using NGS data. The figure illustrates the process of identifying and mapping T-DNA insertion sites in the LMO genome using next-generation sequencing (NGS) data. The top section shows the mapping of reads to the plasmid backbone, highlighting the T-DNA region and its flanking sequences. The bottom section demonstrates the integration of T-DNA into the host genome, with mapped reads confirming the precise location and orientation of the T-DNA insertion. This visualization aids in understanding the structural organization of the inserted genetic material within the LMO genome

To confirm the insertion site, NGS data can be submitted for cases in which the inserted gene is located in the plasmid or in the host genome. Methods and results for identifying the insertion site must be explained. This explanation involves describing the specific location and experimental methods used for confirmation and visualization of the results (Fig. 6). If the genomic information is known, trace information from the host distribution agency and sequence information from databases such as NCBI should be provided. The insertion site is indicated as a coordinate relative to the host. If the genomic information is unknown, at least 500 bp of sequence information should be provided on both sides of the insertion site. Additionally, evidence such as T-DNA junction PCR sequences should be provided. PCR amplification results from the insertion site should be submitted, including a diagram depicting the method.

To evaluate the copy number, detailed analysis methods and criteria must be provided along with an in silico conclusion. A sufficient length of approximately 100 bp is necessary for analysis, to detect junction sequences. The validity of the read depth should be described, and if discarding junction reads, specific details and rationale for this action should be provided. Copy number determination can be confirmed by the number of unique junctions of the T-DNA along with adjacent genomic sequences. The method should be briefly explained, and relevant references should be cited from papers or websites. All detectable copy numbers of introduced DNA, along with chromosome number and location, must be specified. Sequences should be compared with the transformation plasmid and mapped, and this information should be visualized for presentation.

To confirm the copy number and T-DNA sequence at insertion sites, amplicons generated via PCR can be sequenced using NGS. Reads generated from duplicated amplicons are aligned to the reference sequence, and matching segments are extracted. The aligned sequences between the reference and the transformation plasmid are displayed for visualization, along with a coverage graph. Homology with known genes encoding toxins or antinutrients, depending on the nucleotide sequence structure and function of the introduced gene, should be verified. The strategy, software, and all relevant parameters (including algorithms if specified within the software) used for identification must be reported. The version and/or access date of the database should be provided. The method should be briefly explained, and relevant references should be cited from papers or websites. The chromosome number, position, and copy number should be provided to confirm the copy number at the insertion sites.

**Fig. 7:**
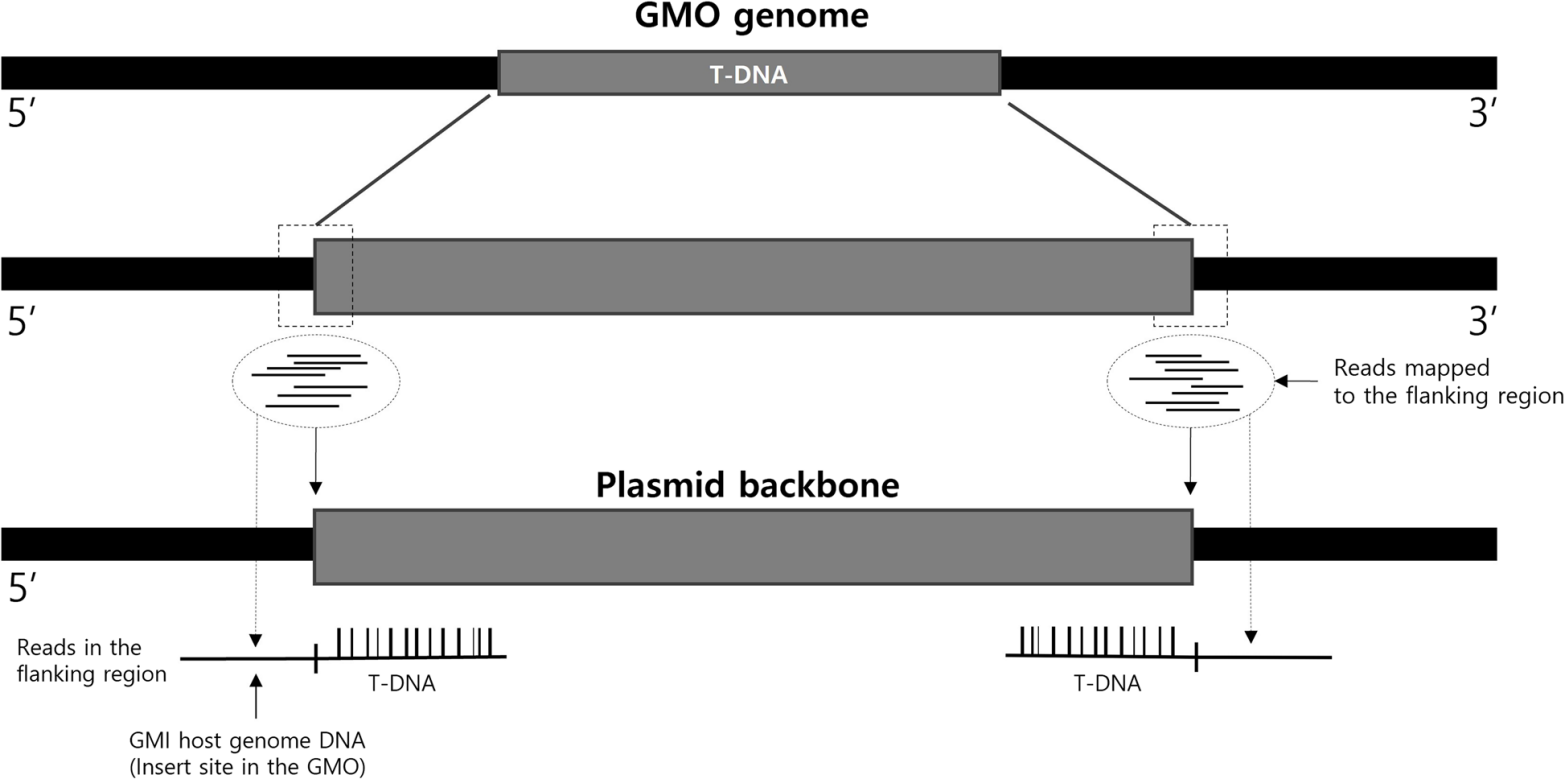
Example of T-DNA insertion site and adjacent flanking region. This figure illustrates the mapping of T-DNA insertion sites and their adjacent flanking regions in both the GMO genome and the plasmid backbone using next-generation sequencing (NGS) data. The top section displays the location of T-DNA within the GMO genome, while the bottom section shows the location of T-DNA within the plasmid backbone. Reads mapped to the flanking regions provide detailed information on the exact insertion sites and orientation of the T-DNA. This visualization is crucial for understanding the structural organization of inserted genetic material

Methods used for adjacent sequence determination (e.g., NGS) and analysis of adjacent sequences (e.g., BLAST) should be described, and evidence data and results should be provided with explanatory text and visual images (Fig. 7). Sequence information for PCR amplification fragments corresponding to each T-DNA junction, i.e., flanking regions, should be provided as evidence. At least 100 bp of sequence information should be provided on both sides of the insertion site and the surrounding region of the introduced gene insertion site. The PCR amplification results of fragments for confirming the LMO genome’s position, along with visualized information comparing the sequence of PCR amplification fragments with the LMO genome’s reference sequence, should be submitted, including a figure illustrating the method.

To confirm the stability across generations, the same analysis performed on key characterized generations should be repeated for at least three consecutive generations, rendering and comparing junction sequences to demonstrate stability. The consistency of the number of junction sites and depth of coverage across multiple generations, along with the absence of a backbone, confirms the stability of the method. Visualization data generated by mapping sequence reads to the transformation plasmid must be compared.

Submitted data should include raw reads in FASTQ format (http://www.bioinformatics.babraham.ac.uk/projects/fastqc/) and sequence alignment information in SAM (Sequence Alignment/Map), BAM (Binary Alignment/Map) [25], and CRAM formats (https://www.ebi.ac.uk/ena/software/cram-toolkit). Alignments to the transformation plasmid should be provided in BAM/SAM format alongside visualization using programs such as IGV. The reliability of each method and its results should be supported by referencing scientific articles published in journals indexed in the Science Citation Index (SCI, SCIE) or relevant websites.

## Discussion

NGS data have been used for whole-genome characterization studies across various fields. For the evaluation of GMO genome characteristics, the use of NGS data was first reported by Kovalic D. et al. [23]. In that study, molecular characterization of the entire genome of the typical GM soybean [*Glycine max* (L.) Merr.] was conducted, providing an equivalent molecular characterization analysis to that of Southern blot-based methods. Furthermore, it has been demonstrated that next-generation sequencing and bioinformatics offer efficiency and consistency in methods compared to the current Southern blot approach. Specifically, investigations of events, including multiple insertion DNA rearrangements, have proven effective in identifying complex cases. Guo B. et al. [13] also demonstrated that WGS is a cost-effective and rapid approach for detecting T-DNA insertions and flanking sequences in soybeans. Similarly, in their study, Guttikonda S. K. et al. [14] demonstrated the effective use of both whole-genome sequencing and target capture sequencing methods to analyze single and stacked transgenic events in soybeans. They asserted that the application of NGS techniques, such as whole-genome sequencing and targeted capture sequencing, in the molecular assessment of transgenic events allows for comprehensive responses to key regulatory inquiries regarding transgene copy number, T-DNA integrity, the stability of T-DNA insertions across different generations, and the presence or absence of plasmid backbone sequences.

In addition, Yang et al. [49] used Illumina HiSeq 2000 equipment to generate 90-bp paired-end sequencing data with an average fragment size of approximately 500 bp, enabling the identification of T-DNA sequences and insertion DNA locations in the whole genome of rice GM events. Park D. et al. [35] also employed transgenic rice and molecular characterization methods based on next-generation sequencing (NGS) using bioinformatics tools. They detected precise insertion locations, copy numbers of transferred DNA, genetic rearrangements, and the absence of backbone sequences, which were equivalent to the results obtained from Southern blot analyses.

Furthermore, Zastrow-Hayes G. M. et al. [50] demonstrated the replacement of Southern blot techniques with NGS data for examining T-DNA in GM maize events. Southern blot analysis is time-consuming and relatively costly and may not provide detailed sequence-level information. To address this issue, a sequence-based technique called Southern-by-Sequencing (SbS), which combines next-generation sequencing (NGS) technology with sequence capture, has been developed as a replacement for Southern blot analysis for event selection in high-throughput molecular characterization environments. It has been demonstrated to be a powerful event screening tool capable of handling molecular characterization environments, providing information on the number of inserted gene loci, copy number, rearrangements, cleavages, or deletions of intended inserted DNA, and the presence of the backbone sequence of the transformation plasmid. Cade R. et al. [3] also demonstrated that whole-genome sequencing results for genetically modified maize have at least the same sensitivity as Southern blot analysis in determining the insert copy number and the presence of unintended insertions and for characterizing small fragment insertions.

Zhang R. et al. [51] analyzed the molecular characterization of transgene integration in transgenic cattle through NGS, demonstrating a reliable and precise method for characterizing transgene sequences, integration sites, and copy numbers in transgenic organisms.

Nevertheless, potential regulatory hurdles or acceptance issues may arise during the transition from traditional methods to NGS. To address these issues, it is necessary to specify and standardize the regulations and submission requirements for NGS-based assessments. For instance, it is crucial to standardize the appropriate amount of NGS data [41, 49] and sequencing coverage depth needed for the molecular characterization of GM events. Sequencing coverage can vary significantly from 10 × to over 75 × depending on the analysis method [50, 23, 14, 26, 20]. While there is debate about the appropriate range for detecting the presence of inserts, several studies have shown that higher sequencing depths are more advantageous for achieving accurate molecular characterization of GM events [1, 46]. However, higher coverage can also increase costs, so it is essential to establish appropriate criteria for plants and microorganisms.

Furthermore, while Southern blot analysis allowed for the submission of photographic evidence of marker bands on gels, NGS faces challenges regarding how to submit evidence. To resolve this issue, guidelines should be provided for submitting not only raw data but also visual materials alongside NGS results, leveraging findings from prominent research studies.

Recent studies have presented evidence that the use of NGS data allows for efficient and reliable molecular characterization of GM crops, potentially replacing or complementing conventional methods. NGS technology offers rapid and efficient protocols for detecting the precise copy number of inserted genes, their genomic locations, the presence of vector backbones, and the stability of T-DNA across generations. Additionally, NGS is sensitive enough to identify nucleotide base substitutions beyond SNPs, including small insertions and deletions, enabling comparative studies across events and reference genomes. The application of new technologies based on scientific data is expected to play a crucial role in increasing consumer confidence.

## Data Availability

No datasets were generated or analysed during the current study.

## Availability of data and materials

No datasets were generated or analysed during the current study.
